# Genome-Wide Identification of DUF26 Domain-Containing Genes in Dongxiang Wild Rice and Analysis of Their Expression Responses under Submergence

**DOI:** 10.3390/cimb44080231

**Published:** 2022-07-27

**Authors:** Cheng Huang, Dianwen Wang, Hongping Chen, Wei Deng, Dazhou Chen, Ping Chen, Jilin Wang

**Affiliations:** Rice National Engineering Research Center (Nanchang), Rice Research Institute, Jiangxi Academy of Agricultural Sciences, Nanchang 330200, China; chenghuang@webmail.hzau.edu.cn (C.H.); dianwen1989@126.com (D.W.); 13970920363@139.com (H.C.); dw19710330@163.com (W.D.); cdz288@163.com (D.C.)

**Keywords:** Dongxiang wild rice (*Oryza rufipogon* Griff.), DUF26 domain, expression mode, submergence tolerance

## Abstract

The DUF26 domain-containing protein is an extracellular structural protein, which plays an important role in signal transduction. Dongxiang wild rice (*Oryza rufipogon* Griff.) is the northern-most common wild rice in China. Using domain analysis, 85 DUF26 domain-containing genes were identified in Dongxiang wild rice (DXWR) and further divided into four categories. The DUF26 domain-containing genes were unevenly distributed on chromosomes, and there were 18 pairs of tandem repeats. Gene sequence analysis showed that there were significant differences in the gene structure and motif distribution of the DUF26 domain in different categories. Motifs 3, 8, 9, 13, 14, 16, and 18 were highly conserved in all categories. It was also found that there were eight plasmodesmata localization proteins (PDLPs) with a unique motif 19. Collinearity analysis showed that DXWR had a large number of orthologous genes with wheat, maize, sorghum and zizania, of which 17 DUF26 domain-containing genes were conserved in five gramineous crops. Under the stress of anaerobic germination and seedling submergence treatment, 33 DUF26 domain-containing genes were differentially expressed in varying degrees. Further correlation analysis with the expression of known submergence tolerance genes showed that these DUF26 domain-containing genes may jointly regulate the submergence tolerance process with these known submergence tolerance genes in DXWR.

## 1. Introduction

Signal transduction mechanisms exist widely in plants and other eukaryotes, and are involved in the regulation of cell functions, the coordination of cell–cell, and the exchange of information between cells and the environment. Proteins with extracellular domains and large gene families encoding secretory proteins in plants play an important role in sensing environmental changes and development through signal transduction mechanisms [1,2,3]. Receptor like kinase (RLK) with an extracellular domain is involved in signal sensing, while intracellular kinase domain transduces signal to substrate protein. RLK plays an important role in stress response, hormone signal transduction, cell wall monitoring, and plant development.

The DUF26 domain (PF01657) belongs to the extracellular domain, and its core contains a conserved cysteine motif (C-8X-C-2X-C), which exists in three plant proteins [4,5]. The first is a cysteine rich receptor like secreted protein (CRRSP). The most typical CRRSP is Gnk2 from Ginkgo biloba leaves, which has an antifungal activity as mannose binding lectin in vitro [5]. Two maize CRRSPs have also been shown to bind mannose and participate in the defense against fungal pathogens [6]. The second is a cysteine rich receptor kinase (CRK) that has a typical DUF26 structure in the extracellular region, forming a large RLK subgroup in plants and playing a role in the response of Arabidopsis to stress [7,8,9,10,11,12,13]. The third DUF26 domain-containing protein is plasmodesmata localized protein (PDLP). PDLP contains two DUF26 domains and a transmembrane helix in its extracellular domain, but lacks a kinase domain. They are related to plasmodesmata and participate in intercellular signal transduction, pathogen response, systemic signal transduction, and callose deposition control [14,15]. However, the specific biochemical function of the DUF26 domain-containing gene in plants is still unclear.

Rice is one of the most important food crops in the world, and nearly half of the world’s population lives on rice [16]. Submergence stress is one of the most serious natural disasters facing mankind, and has become an important factor limiting the stable yield and yield increase of rice [17]. DXWR is a common wild rice with the highest latitude and the northernmost distribution, which is rich in disease and insect resistance genes and submergence, cold, drought, and barren resistance genes [18]. Previous studies have shown that the DUF26 domain-containing gene plays a role in signal transduction and stress response. However, the research on the molecular mechanism of the DUF26 domain-containing gene response to submergence stress in DXWR is still very limited. The whole genome recognition and characterization of DUF26 domain-containing genes in DXWR is helpful to better explore the molecular mechanism of biotic or abiotic stress resistance. The high-quality sequencing of DXWR has been completed, which provides an excellent opportunity for us to identify and dissect the DUF26 domain-containing genes of DXWR. In this study, the DUF26 domain-containing gene in DXWR were comprehensively studied including phylogeny, chromosome location, gene structure, protein motif, evolution, expression pattern analysis, and correlation analysis of submergence stress. In general, the systematic analysis of DUF26 domain-containing proteins and their expression regulation mode under submergence tolerance has laid the foundation for further exploring the function of DUF26 domain-containing genes in DXWR.

## 2. Materials and Methods

### 2.1. Identification of DUF26 Domain-Containing Genes in DXWR

The sequence information of DXWR comes from the gene annotation website (http://www.ricegermplasmgenome.com/, accessed on 20 May 2022), and the hidden Markov model of DUF26 domain-containing genes (PF01657) was used to search and screen candidate genes in hmmer3 [19,20]. Then, it was passed through the NCBI conservative domain database (CDD; https://www.ncbi.nlm.nih.gov/cdd, accessed on 26 May 2022) and SMART database (http://smart.embl-heidelberg.de/, accessed on 12 June 2022) to confirm the integrity of the protein domain [21,22]. On this basis, pkinase_Tyr (PF07714) searched for proteins containing kinase domains, and identified candidate protein domains through the PFAM, SMART, and NCBI-CDD protein databases. The isoelectric point and molecular weight of the DUF26 domain-containing genes were obtained from the ExPASY website [23]. The online analysis software Wolf Psort (http://wolfpsort.hgc.jp/, accessed on 16 June 2022) was used to predict the subcellular localization of the DUF26 domain-containing protein in DXWR.

### 2.2. Analysis of the Main Characteristics of DUF26 Domain-Containing Genes in DXWR

Using MEGA11, the phylogenetic tree of DXWR was constructed by neighbor joining (NJ) and bootstrap repeats set to 1000 [24]. The distribution of all DUF26 domain- containing genes on the chromosome of DXWR was analyzed and visualized by TBtools [25]. The characteristic motif of the DUF26 domain-containing gene was determined by MEME (http://meme-suite.org/tools/meme, accessed on 12 June 2022), the base sequence number was 20, and the sequence of site distribution was 0 or 1 occurrence for each sequence [26].

### 2.3. Repeat Events and Collinearity Analysis of DUF26 Domain-Containing Genes

The identification of serial repeat events was analyzed by the multilinear analysis tool MCscanX [27]. The genome data of Wheat(Triticum_aestivum.IWGSC.52), Sorghum(Sorghum_bicolor.Sorghum_bicolor_NCBIv3.52), and Maize(Zea_mays.Zm-B73- REFERENCE-NAM-5.0.52) were all from the Ensembl Plant genome website (https://plants.ensembl.org/index.html, accessed on 20 June 2022) and Zizania(Zlat_genome_v1) was downloaded from the website (https://ngdc.cncb.ac.cn/, accessed on 20 June 2022). The colinear relationship between DXWR and the gramineous crops was analyzed by JCVI [28].

### 2.4. Expression Data of DUF26 Domain-Containing Genes under Submergence

The expression data of all genes were from the transcriptome data of submergence stress tolerance (unpublished), in which AG is anaerobic germination, SS is seedling submergence, and CK is normal growth. Anaerobic germination and normal germination treatment: the dxwr seeds were shelled, then germinated in 20 cm deep and 5 mm normal water, respectively, and then the embryo tissues germinated for 2D and 4D were taken successively. Submergence treatment at the seedling stage: the seedlings were normally grown to the leaf stage (6LS), and all of them were submerged in the deep water of 50 cm. The aboveground tissues of 0, 3, and 5 days of submergence were taken, respectively. The aboveground tissues included the stem base area of 5 mm in the area of the internode, node, stem tip, and leaf base. All of the above materials were grown in incubators under the conditions of 28 °C normal temperature, 14 h of light, and 10 h of darkness. All samples were quickly frozen in liquid nitrogen and stored at −80 °C until the RNA was extracted (in order to avoid the difference of gene expression between samples caused by circadian rhythm, submergence treatment, and sample collection were conducted at 4 pm).

### 2.5. Correlation Analysis between DUF26 Domain-Containing Genes and Submergence Tolerance Genes

In order to predict the function of DUF26 domain-containing genes, submergence tolerant genes with known functions include *RAmy3D* (*LOC_Os08g36910*), *CIPK14/15* (*LOC_Os12g02200/LOC_Os11g02240*), *G3PDH* (*LOC_Os02g38920*), *MPK3* (*LOC_* *Os03g17700*), *OsERF66/67* (*LOC_Os03g22170/LOC_Os07g47790*), *PPDK* (*LOC_* *Os03g31750*), *SLR1* (*LOC_Os03g49990*), *SLRL1* (*LOC_* *Os01g45860*), and *SnRK1A* (*LOC_* *Os05g45420*). The correlation analysis between the DUF26 domain-containing genes and submergence tolerant genes for functional identification was calculated using the cor function in R language and default Pearson correlation coefficient.

## 3. Results

### 3.1. Identification of DUF26 Domain-Containing Genes in DXWR

Hmmer3 was used to search all of the DUF26 domain-containing genes in the local DXWR database. After de-redundancy, candidate DUF26 domain-containing genes were obtained. Finally, 85 DUF26 domain-containing genes were identified, of which 10 did not contain the pkinase-cysteine rich receptor like secretory proteins (CRRSPs) of the Tyr domain and 24 plasmodesmata localization proteins (PDLPs), 51 of which contain pkinase-cysteine rich receptor protein kinase (CRKs) of the Tyr domain. The cysteine rich receptor protein kinases (CRKs) can be further divided into two categories: the cysteine rich receptor protein kinases (sdCRKs) containing a single DUF26 domain and the cysteine rich receptor protein kinases (ddCRKs) containing double DUF26 domains are 6 and 45, respectively (Table S1). In addition, the physical and chemical properties of 85 DUF26 domain-containing genes including the molecular weight, isoelectric point, gene location on chromosome, and subcellular localization were also analyzed (Table S1).

### 3.2. Chromosomal Localization of DUF26 Domain-Containing Genes in DXWR

**Figure 1:**
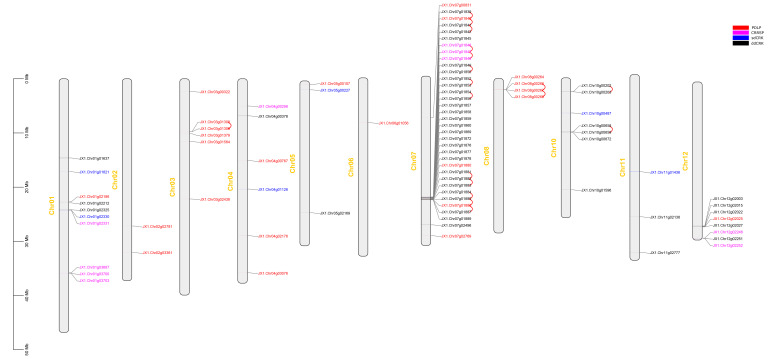
The distribution of DUF26 domain-containing genes on chromosomes. Different colors represent different types of DUF26 containing genes, in which red represents PDLPs, magenta represents CRRSPs, blue represents sdCRKs, black represents ddCRKs, and the red connecting line represents tandem repeat gene pairs.

The chromosomal mapping analysis showed that 85 DUF26 domain-containing genes were present on all chromosomes except for chromosome 9. The results showed that the DUF26 domain-containing genes were unevenly distributed on the chromosomes, with only one gene on Chr6 and 35 genes on Chr7. It is worth noting that those belonging to the same group in the phylogenetic tree were not mapped on the same chromosome, but scattered in different positions on the same chromosome or exist on different chromosomes (Figure 1). In addition, the genome collinearity analysis of DXWR showed that 18 pairs of DUF26 domain-containing genes were tandem repeats, indicating that tandem repeats played an important role in the expansion of DUF26 domain-containing genes (Figure 1).

### 3.3. Motifs and Structural Composition of DUF26 Domain-Containing Genes in DXWR

**Figure 2:**
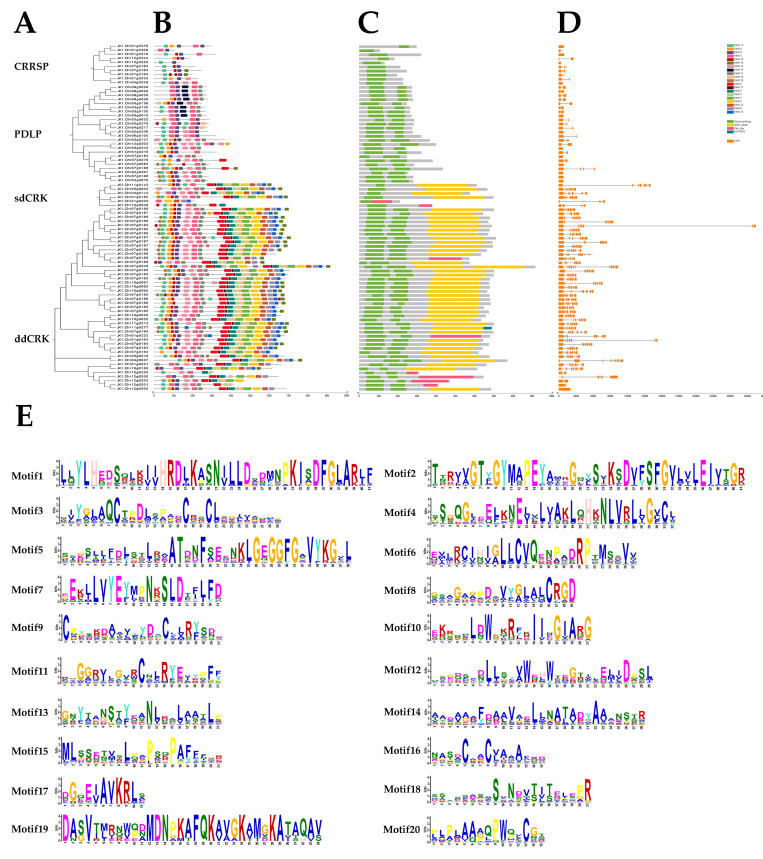
The phylogenetic relationship, structure, domain, and conserved motif analysis of DUF26 domain-containing genes in DXWR. (**A**) The phylogenetic tree was constructed based on the full-length sequences of DXWR DUF26 domain-containing proteins using MEGA12. (**B**) The distribution of motifs of the MAPK proteins. The conserved motifs of DUF26 domain-containing proteins were determined by MEME and visualized by TBtools. (**C**) Domain analysis of 85 DUF26 domain-containing proteins. Green represents DUF26 domain (stress antifung). Yellow represents the sTKc_IRAK kinase domain. Magenta represents the PKc_like domain. Blue green represents the duf3404 domain. (**D**) The exon–intron structures of the DUF26 domain-containing genes. Orange boxes indicate exons. (**E**) The sequence logo of the DUF26 domain-containing proteins motifs. The height of each amino acid represents the relative frequency of the amino acid at that position.

The 85 DUF26 domain-containing genes were divided into four groups, and the phylogenetic tree was constructed by using amino acid sequences. At the same time, we analyzed the conserved motifs of the DUF26 domain-containing genes to explore various biological functions of the protein domain. Twenty conserved motifs were identified, among which Motif 3, 8, 9, 13, 14, 16, and 18 were conserved in four groups of DUF26 domain-containing genes (Figure 2). Motifs 11 and 20 only appeared in three other DUF26 domain-containing genes except for CRRSPs. Meanwhile, 47 of the 51 cysteine rich receptor protein kinases (CRKs) contained conserved Motifs 1, 2, 4, 5, 6, 7, 10, 12, 15, and 17. In addition, Motif 19 was found only in eight plasmodesmata localization proteins (PDLPs). Moreover, the DUF26 domain-containing gene was also analyzed, and it was found that the location of this domain was highly consistent with that of the motif. Specifically, the DUF26 domain-containing genes in members of the same class had similar conserved motifs, and the motifs of the members of different classes were quite different. Whether these motif composition differences affect their biological functions needs to be further studied through biological experiments (Figure 2). At the same time, the exon/intron patterns of 85 DUF26 domain-containing genes were also studied (Figure 2). The results showed that the number of exons of the DUF26 domain-containing gene ranged from 1 to 11, and 74 of them contained at least two exons. The gene structure of the DUF26 domain-containing in the CRKs group was more complex than that of PDLPs and CRRSPs.

### 3.4. Collinearity Analysis of DUF26 Domain-Containing Genes in DXWR among Gramineous Species

**Figure 3:**
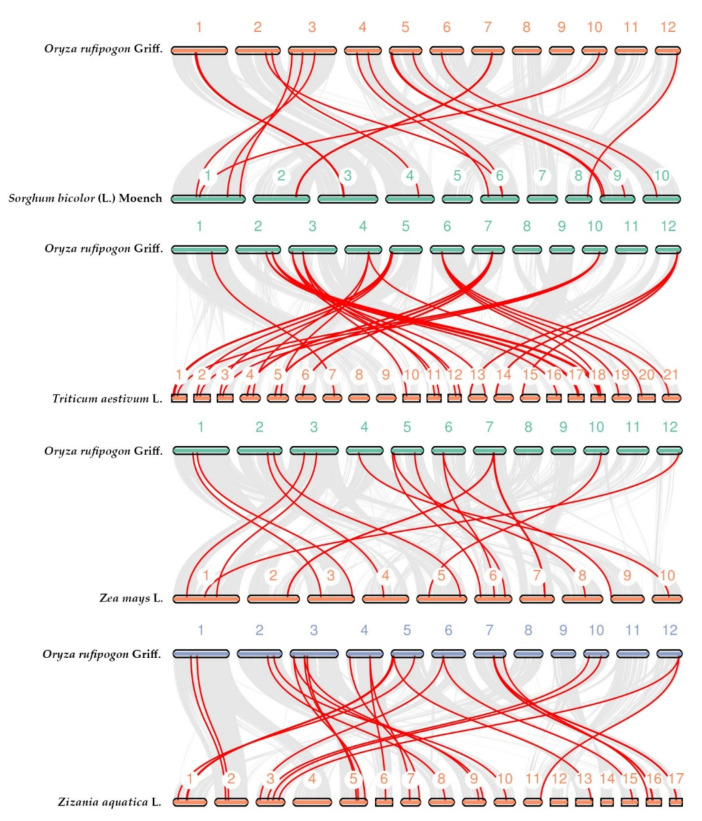
The collinearity analysis of the DXWR and gramineaes plants. Arabic numerals indicate chromosome numbers. The red line highlights the collinearity gene pairs containing the DUF26 domain-containing genes among different species.

The collinearity between species provides a reference for studying the evolution of gene families and gene functions. Therefore, the collinearity of the DUF26 domain-containing genes between DXWR and four other gramineous plants was analyzed (Figure 3). The collinearity analysis showed that DXWR and wheat had the most homologous genes, with 64 pairs, followed by zizania (41 pairs), sorghum (27 pairs), and maize (23 pairs). Orthologous DUF26 domain-containing genes in five gramineous crops (Table S2). The 17 domain-containing genes containing *JX1.Chr01g02212*, *JX1 Chr02g02781*, *JX1 Chr02g03361*, *JX1 Chr03g01308*, *JX1 Chr05g00107*, *JX1 Chr05g00227*, *JX1 Chr06g01056*, *JX1 Chr07g01839*, *JX1 Chr07g01841*, *JX1 Chr07g01842*, *JX1 Chr07g01845*, *JX1 Chr07g01847*, *JX1 Chr07g01849*, *JX1 Chr07g01853*, *JX1 Chr07g01869*, *JX1 Chr10g01596,* and *JX1.Chr12g02022* were highly conserved among the five gramineous species. Among them, 11 genes had three or more homologous genes in wheat. These highly conserved homologous genes are of great significance in exploring the relationship between species and predicting gene function.

### 3.5. Expression Analysis of DUF26 Domain-Containing Genes in DXWR under Submergence

**Figure 4:**
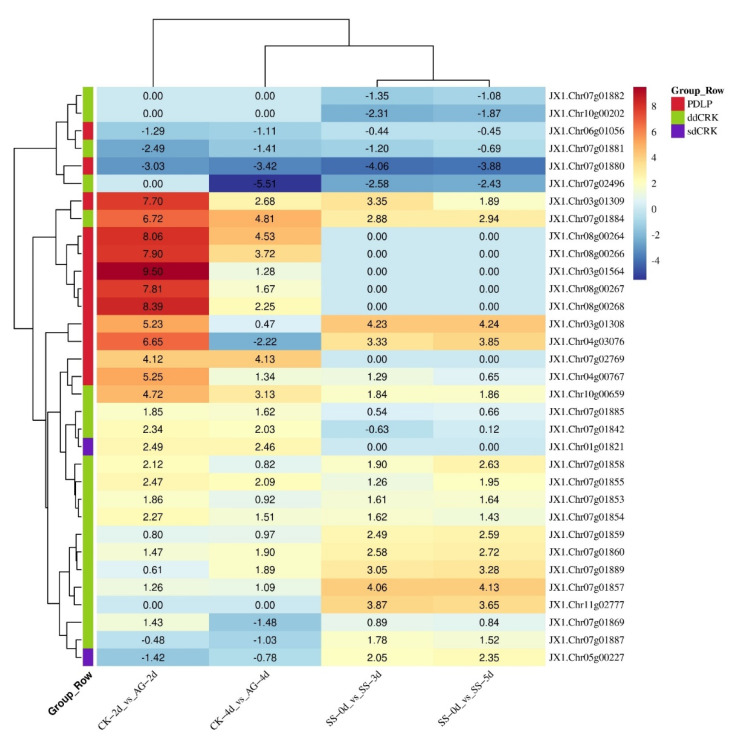
The expression profile of the DUF26 domain-containing genes after different periods of submergence, in which AG is anaerobic germination, SS is seedling submergence, and CK is normal growth. The heatmap was generated by taking log2 fold of the FPKM ratio.

In order to explore the response of the DUF26 domain-containing gene in DXWR to submergence, the transcriptome data of the DUF26 domain-containing genes before and after submergence germination and seedling submergence treatment were studied. The results showed that 33 out of the 85 DUF26 domain-containing genes in DXWR responded to submergence (Figure 4). A total of 50% (12/24) of PDLPs were induced to express by submergence including *JX1.Chr06g01056* and *JX1.Chr07g01880*, which were down-regulated after submerged germination and seedling submergence treatment, while the other 10 PDLPs were significantly upregulated after 2 days of submerged germination, but the upregulation multiple decreased after 4 days of submerged germination. The expression of *JX1.Chr03g 01309*, *JX1.Chr03g01308*, and *JX1.Chr04g03076* was upregulated after 3 and 5 days of seedling submergence. At the same time, 42% (19/42) of ddCRKs responded to submergence. *JX1.Chr07g01882*, *JX1.Chr10g00202*, *JX1.Chr07g01881*, and *JX1.Chr07g02496* were downregulated in both the submerged germination and seedling submergence treatments, while *JX1.Chr07g01884* was upregulated in both the submergence treatments. In addition, there were 10 ddCRKs with different responses under the two submergence treatments, for example, *JX1.Chr07g01857* and *JX1.Chr11g02777* were upregulated after submergence treatment at the seedling stage, but downregulated at the germination stage.

### 3.6. Correlation Analysis between DUF26 Domain-Containing Genes and Submergence Tolerant Response Genes in DXWR

**Figure 5:**
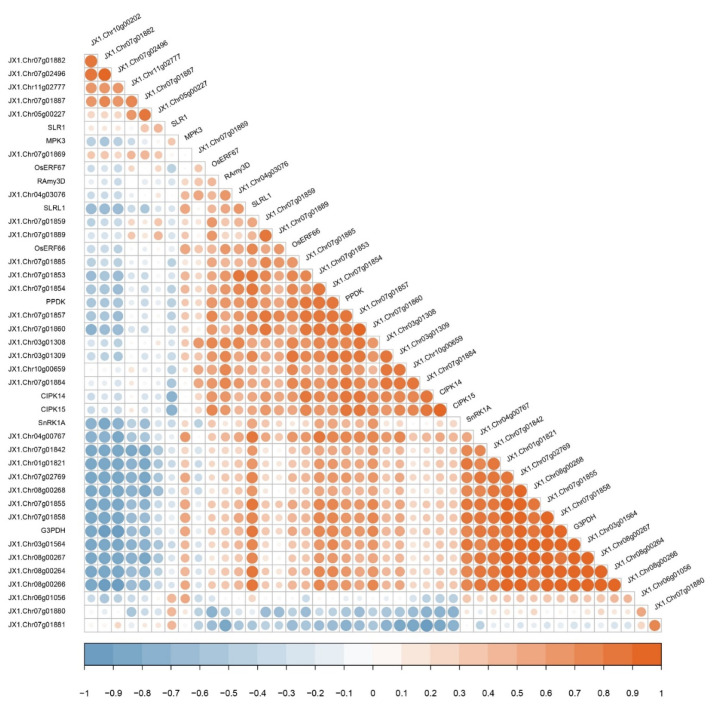
The correlation analysis of the DUF26 domain-containing genes and submergence tolerance genes in DXWR.

In order to further study whether the DUF26 domain-containing genes are involved in the response of DXWR to submergence, the correlation between 33 DUF26 domain-containing genes differentially expressed in submergence germination and seedling submergence treatment and other known genes involved in the response of rice to submergence were analyzed. The expression data of all genes were used under submergence stress (Table S3). The results showed that 11 DUF26 domain-containing genes including *JX1.Chr07g01842*, *JX1.Chr01g01821*, *JX1.Chr08g00268*, *JX1.Chr03g01564*, and *JX1.Chr03g01564* were significantly positively correlated with the expression levels of SnRK1A and G3PDH. In addition, 13 DUF26 domain-containing genes including *JX1.Chr07g01885*, *JX1. Chr07g01857*, and *JX1.Chr07g01860* were significantly positively correlated with the expression levels of *CIPK14/15*, *PPDK*, *OsERF66/67*, *RAmy3D*, and *SLRL1* (Figure 5). In conclusion, the correlation analysis showed that the 33 DUF26 do main-containing genes were involved in the response of DXWR to submergence and were likely to be related to known submergence tolerance genes.

## 4. Discussion

The DUF26 domain-containing genes are an ancient family in plants, which mainly regulate plant growth, development, and respond to various biological and abiotic stresses through signal transduction. DUF26 domain-containing genes have been identified in a variety of plants, where a total of 108, 79, and 48 DUF26 domain-containing genes have been identified in Arabidopsis, rice, and maize, respectively [29]. We identified 85 DUF26 domain-containing genes in the genome of DXWR (Table S1). According to the differences in domains, they were divided into four categories, in which the number of CRRSPs, PDLPs, sdCRKs, and ddCRKs were 10, 24, 6, and 45, respectively. Tandem repeat genes play a role in processes that require rapid adaptation such as adaptation to the environment, pathogen response, and secondary metabolism [30]. It was found that the DUF26 domain-containing genes in DXWR were mainly clustered on chromosomes, and there were 18 pairs of tandem repeat genes, which played an important role in the expansion of its members. For example, *JX1.Chr08g00266*, *JX1.Chr08g00267*, and *JX1.Chr08g00268* are three genes derived from tandem repeats. Interestingly, they are significantly upregulated during anaerobic germination, indicating that these three genes may participate in the anaerobic germination process together.

Orthologous genes are highly conserved among species, and they may be preserved due to their similar important functions in the process of evolution [31]. As a hexaploid plant, wheat has experienced two polyploidization events. Theoretically, one DXWR gene corresponds to three wheat homologous genes [32]. For example, *JX1.Chr07g01849* and 11 other genes have three homologous genes in wheat, indicating that these genes are very conservative in the evolution of wheat. In addition, *JX1.Chr07g01882* and *JX1.Chr12g02022* have six homologous genes in wheat, indicating that these two genes have been expanded in the process of wheat evolution. Rice, sorghum, and maize have experienced at least two whole genome duplication events before the differentiation of Gramineae [32]. Theoretically, there are duplicate copies of DUF26 domain-containing genes among different species. For example, *JX1.Chr07g01849* and *JX1.Chr06g01056* have two lineal homologous genes in sorghum and maize, respectively. Zizania has good characters of deep water tolerance, and there is a significant collinearity between the zizania and rice genome [33]. There are 35 DUF26 domain-containing genes in DXWR that are collinear with zizania, of which about 23% (8/35) have two orthologs genes in zizania. In the collinearity analysis with wheat, maize, sorghum, and zizania, 17 DUF26 domain-containing genes were found to be highly conserved in five gramineous crops, indicating that they may play similar functions. For example, *JX1.Chr03g01308* is highly conserved in five gramineous crops, and it is significantly upregulated during the anaerobic germination and seedling submergence of DXWR, suggesting that this gene may play a similar role in the submergence tolerance of these species. In addition, about 47% (40/85) of the DUF26 domain-containing genes in DXWR have no corresponding ortholog genes in other gramineous crops, and these genes may play a unique role in the evolution of DXWR and be preserved. For example, *JX1.Chr04g00767*, a unique DUF26 domain-containing gene in DXWR, was significantly upregulated during anaerobic germination and seedling flooding, suggesting that it may be the reason why DXWR has strong submergence tolerance.

The analysis of gene structure shows that there were similar intron/exon arrangement patterns and numbers among the genes located in the same group of intron DUF26 domain, and their conservative domains also had similar numbers and position orders, indicating that the members of the subfamily were relatively conservative in the process of evolution, but there are great structural differences among members of different groups, where different cleavage patterns may play an important role in the functional differentiation of DUF26 domain-containing genes. Protein sequence analysis showed that all DUF26 domain-containing genes contained highly conserved Motif 3, 8, 9, 13, 14, 16, and 18, and had similar motif structures among the same group of DUF26 domain-containing genes. Similarly, the unique motif provides an idea for studying the function of DUF26 domain-containing genes. For example, Motif 19 only appeared in eight PDLPs. It is noteworthy that these eight genes were significantly upregulated during anaerobic germination. Therefore, it is speculated that Motif 19 may play an important role in the anaerobic germination of DXWR, which still needs to be verified by subsequent experiments.

There are two strategies in the process of rice submergence tolerance: the first one is to avoid submergence, which shows coleoptile elongation in the anaerobic germination stage, rapid stem elongation in the seedling stage, and the second one is to stop growth in the seedling stage [34]. Prior to this study, there was no report on the role of the DUF26 domain-containing gene in submergence tolerance. This study confirmed that 33 of the 85 DUF26 domain-containing genes in DXWR responded to anaerobic germination or seedling submergence to varying degrees. Subsequent correlation analysis showed that these response genes may have the same expression pattern as the known submergence tolerant genes. Protein kinase CIPK14/15 plays an important role in the tolerance of rice to hypoxia stress. SnRK1A is an important intermediary in the sugar signal cascade. Under submergence conditions, CIPK15 links the hypoxia signal with the SnRK1A dependent sugar signal cascade, regulates the production of sugar and energy, and enables rice to grow under submergence conditions [35,36]. Similarly, G3PDH plays a key role in glycolysis [37]. MPK3 is induced by submergence and plays a key role in its adaptation to submergence conditions [38]. *JX1.Chr07g01842*, *JX1.Chr01g01821*, *JX1.Chr08g00268*, *JX1.Chr03g01564*, *JX1.Chr08g00267*, *JX1.Chr08g00264*, *JX1.Chr08g00266*, *JX1.Chr07g02769*, *JX1.Chr07g01855*, and *JX1.Chr07g01858* are the 10 genes highly correlated with the *SnRK1A* and *G3PDH* expression regulation of the sugar signal channel, and also positively correlated with *CIPK14/15* and *MPK3*. Interestingly, these genes were highly upregulated during anaerobic germination, indicating that they may participate in the anaerobic germination process of DXWR by affecting glucose metabolism. Pyruvate phosphate double kinase gene (*PPDK*) is a response gene to hypoxia stress. Hypoxia stress induces the expression of PPDK in the rice roots, leaf sheaths, and shoots of etiolated seedlings [39]. Under anoxic conditions, the α-amylase gene (*RAmy3D*) is upregulated, which is very important for germination and growth under hypoxia [40,41,42]. *OsERF66/67* is stable under hypoxia. The overexpression of *OsERF66/67* will lead to the increased expression of anaerobic survival genes, indicating that they are responsible for submergence mediated transcriptional regulation in submergence tolerance strategies [43]. Two GRAS proteins SLR1/SLRL1 are inhibitors of GA signal, which mediate submergence tolerance by limiting gibberellin response in rice [44,45,46]. *JX1.Chr07g01885*, *JX1.Chr07g01857*, *JX1.Chr07g01860*, *JX1.Chr07g01859*, *JX1.Chr07g01889*, *JX1.Chr03g01308*, *JX1.Chr03g01309*, *JX1.Chr07g0188*, *JX1.Chr10g0065*, *JX1.Chr04g00767*, *JX1.Chr07g01853*, and *JX1.Chr07g01854* had a highly similar expression and regulation pattern with *PPDK*, *OsERF66/67*, *RAmy3D*, and *SLRL1*, except for *JX1.Chr07g01885*, which was upregulated only in anaerobic germination, and the rest were upregulated in the process of seedling submergence. These results suggest that these genes may play an important role in the submergence tolerance of DXWR.

## 5. Conclusions

In this study, 85 DUF26 domain-containing genes were identified from the DXWR genome. These genes were unevenly distributed on 12 chromosomes and had the characteristics of cluster arrangement. According to the composition of the conserved domains, the DUF26 domain-containing genes in DXWR can mainly be divided into four categories: CRRSPs, PDLPs, sdCRKs, and ddCRKs. The protein sequence analysis showed that DUF26 domain-containing genes in the same group had a similar structure and motif composition. The collinearity analysis with gramineous crops showed that DUF26 domain-containing genes were preserved and lost during evolution, and some genes with special functions were preserved among species. Transcriptome data analysis showed that 33 DUF26 domain-containing genes responded to anaerobic germination and seedling submergence to varying degrees. Subsequent correlation analysis showed that the involvement of these genes in the submergence tolerance process may be related to the known submergence tolerance genes. The study of the DXWR DUF26 domain-containing gene family in the organization, structure, evolution, and expression level under submergence conditions is conducive to the functional analysis of DUF26 domain-containing genes, and lays an important foundation for a better understanding of the molecular mechanism of submergence tolerance in DXWR.

## Data Availability

Not applicable.

## Supplementary Materials

The following supporting information can be downloaded at: https://www.mdpi.com/article/10.3390/cimb44080231/s1.
