# Supplementary material for: Genome-Wide Identification of DUF26 Domain-Containing Genes in Dongxiang Wild Rice and Analysis of Their Expression Responses under Submergence
Source: Curr Issues Mol Biol. 2022 Jul 27;44(8):3351–63. doi: 10.3390/cimb44080231 (PMC9406443; doi:10.3390/cimb44080231)
Supplement: Supplementary file 1 [file cimb-44-00231-s001.zip › cimb-1795463-supplementary.pdf]

current issues in molecular biology

MDPI

Supplementary

Genome-Wide Identification of DUF26 Domain-Containing Genes in Dongxiang Wild Rice and Analysis of Their Expression Responses under Submergence

Cheng Huang †, Dianwen Wang †, Hongping Chen, Wei Deng, Dazhou Chen, Ping Chen * and Jilin Wang *

Rice National Engineering Research Center (Nanchang), Rice Research Institute, Jiangxi Academy of Agricultural Sciences, Nanchang 330200, China; chenghuang@webmail.hzau.edu.cn (C.H.); dianwen1989@126.com (D.W.); 13970920363@139.com (H.C.); dw19710330@163.com (W.D.); cdz288@163.com (D.C.)

* Correspondence: cp87090379@163.com (P.C.); wangjilin1982@163.com (J.W.); Tel.: +86-185-7906-9996 (P.C.); +86-133-8753-2293 (J.W.)

† These authors contributed equally to this work.

Table S1. Information of DUF26 domain-containing genes in DXWR

Gene name	Group	physical location	PI(pH)	MW	Subcellular localization
JX1.Chr01g01637	ddCRK	Chr1:14609564-14626929(+)	5.93	70964.02	chlo
JX1.Chr01g01821	sdCRK	Chr1:17138528-17141757(+)	5.85	76923.82	golg_plas
JX1.Chr01g02186	PDLD	Chr1:22699570-22701207(-)	5.32	34995.87	extr
JX1.Chr01g02212	ddCRK	Chr1:22898221-22902623(-)	8.2	71101.95	plas
JX1.Chr01g02325	ddCRK	Chr1:24171916-24180115(-)	5.73	76027.8	extr
JX1.Chr01g02330	sdCRK	Chr1:24231103-24239199(-)	5.31	23377.74	chlo
JX1.Chr01g02331	CRRSP	Chr1:24239717-24241680(-)	5.95	25088.26	extr
JX1.Chr01g03687	CRRSP	Chr1:35814005-35814793(-)	4.76	11171.81	extr
JX1.Chr01g03700	CRRSP	Chr1:35930135-35931106(+)	4.92	33390.46	chlo
JX1.Chr01g03703	CRRSP	Chr1:35936308-35937207(+)	4.82	30806	extr
JX1.Chr02g02781	PDLD	Chr2:27184179-27185765(+)	8.63	29243.72	chlo
JX1.Chr02g03361	PDLD	Chr2:32030222-32031058(+)	8.58	27690.04	golg
JX1.Chr03g00322	PDLD	Chr3:2328126-2330110(-)	6.4	29449.49	chlo
JX1.Chr03g01308	PDLD	Chr3:9768347-9769238(-)	8.61	28176.01	extr
JX1.Chr03g01309	PDLD	Chr3:9772379-9773264(-)	8.15	28462.43	extr
JX1.Chr03g01379	PDLD	Chr3:10142571-10144327(+)	8.42	38225.74	plas
JX1.Chr03g01564	PDLD	Chr3:11572584-11574976(-)	8.2	27095.63	chlo
JX1.Chr03g02438	PDLD	Chr3:22192078-22192818(-)	6.32	25550.37	E.R.
JX1.Chr04g00298	CRRSP	Chr4:5034464-5035153(-)	6.53	23628.75	chlo
JX1.Chr04g00378	ddCRK	Chr4:6850023-6861320(+)	5.79	83784.43	plas
JX1.Chr04g00767	PDLD	Chr4:15089664-15090509(-)	8.29	29193.24	extr
JX1.Chr04g01126	sdCRK	Chr4:20353204-20358177(-)	6.47	61837.7	chlo
JX1.Chr04g02176	PDLD	Chr4:28969996-28972690(+)	4.9	28557.12	golg
JX1.Chr04g03076	PDLD	Chr4:35815285-35816055(-)	5.88	27302.16	extr
JX1.Chr05g00107	PDLD	Chr5:683685-684503(-)	5.06	28322.09	extr

JX1.Chr05g00227	sdCRK	Chr5:1640205-1643230(+)	6.68	71769.46	plas
JX1.Chr05g02169	ddCRK	Chr5:24326377-24330114(+)	7.14	72274.73	nucl
JX1.Chr06g01056	PDLD	Chr6:8266378-8269638(-)	8.34	31562.28	E.R.
JX1.Chr07g00831	PDLD	Chr7:7650123-7651056(+)	5.02	30646.62	chlo
JX1.Chr07g01839	ddCRK	Chr7:22288801-22292115(+)	6.39	71271.34	chlo
JX1.Chr07g01840	PDLD	Chr7:22292919-22293662(+)	7.51	27019.68	chlo
JX1.Chr07g01841	ddCRK	Chr7:22316201-22319498(+)	7.15	69130.15	chlo
JX1.Chr07g01842	ddCRK	Chr7:22328460-22334358(+)	5.22	73508.9	plas
JX1.Chr07g01845	ddCRK	Chr7:22401583-22406479(+)	5.89	77038.05	plas
JX1.Chr07g01846	CRRSP	Chr7:22410679-22411807(+)	8.08	26619.49	extr
JX1.Chr07g01847	CRRSP	Chr7:22421369-22422742(+)	8.05	23162.4	extr
JX1.Chr07g01848	CRRSP	Chr7:22425340-22426319(+)	8.6	21018.84	extr
JX1.Chr07g01849	ddCRK	Chr7:22446175-22480736(+)	8.7	74990.66	plas
JX1.Chr07g01850	ddCRK	Chr7:22481149-22484789(+)	8.09	73986.44	plas
JX1.Chr07g01852	ddCRK	Chr7:22488436-22492333(+)	8.07	73694.03	plas
JX1.Chr07g01853	ddCRK	Chr7:22493282-22499452(-)	7.41	75355.25	plas
JX1.Chr07g01854	ddCRK	Chr7:22499602-22502701(+)	7.8	73801.48	plas
JX1.Chr07g01855	ddCRK	Chr7:22505105-22508057(+)	5.53	74139.6	plas
JX1.Chr07g01857	ddCRK	Chr7:22514101-22517191(-)	7.04	76369.05	plas
JX1.Chr07g01858	ddCRK	Chr7:22519666-22522367(-)	6.03	70819	extr
JX1.Chr07g01859	ddCRK	Chr7:22525775-22528918(+)	5.82	73605.04	plas
JX1.Chr07g01860	ddCRK	Chr7:22533850-22536972(+)	6.04	73838.09	plas
JX1.Chr07g01869	ddCRK	Chr7:22620194-22623463(+)	6.57	74089.83	extr
JX1.Chr07g01872	ddCRK	Chr7:22658798-22662812(-)	8.08	74774.3	plas
JX1.Chr07g01876	ddCRK	Chr7:22711696-22720124(-)	8.62	76152	vacu
JX1.Chr07g01877	ddCRK	Chr7:22726297-22729868(-)	6.23	70263.91	cyto
JX1.Chr07g01878	ddCRK	Chr7:22733291-22738306(+)	6.31	77561.11	plas
JX1.Chr07g01880	PDLD	Chr7:22740300-22746688(-)	8.16	46044.99	chlo
JX1.Chr07g01881	ddCRK	Chr7:22750166-22753055(+)	6.36	77168.12	plas
JX1.Chr07g01882	ddCRK	Chr7:22757887-22767471(-)	7.82	74562.45	plas
JX1.Chr07g01883	ddCRK	Chr7:22770968-22774797(+)	6.35	74407.96	plas
JX1.Chr07g01884	ddCRK	Chr7:22783828-22787790(-)	8.13	72189.29	plas
JX1.Chr07g01885	ddCRK	Chr7:22789653-22795525(-)	6.83	69248.99	plas
JX1.Chr07g01886	PDLD	Chr7:22810354-22811202(-)	6.56	29884.76	chlo
JX1.Chr07g01887	ddCRK	Chr7:22828315-22831139(-)	7.01	61638.2	plas
JX1.Chr07g01889	ddCRK	Chr7:22834660-22838506(-)	7.08	63299.09	nucl
JX1.Chr07g02496	ddCRK	Chr7:27452176-27462589(-)	5.25	100372.24	plas
JX1.Chr07g02769	PDLD	Chr7:29465903-29467563(+)	8.25	41786.26	plas
JX1.Chr08g00264	PDLD	Chr8:2076337-2077648(-)	8.19	29694.74	extr
JX1.Chr08g00266	PDLD	Chr8:2081821-2083054(-)	8.14	30385.7	chlo
JX1.Chr08g00267	PDLD	Chr8:2085369-2086566(-)	8.2	29828.83	extr
JX1.Chr08g00268	PDLD	Chr8:2088452-2089392(+)	8.2	29604.61	extr

JX1.Chr10g00202	ddCRK	Chr10:2558402-2561226(-)	7.03	72615.02	vacu
JX1.Chr10g00203	ddCRK	Chr10:2566522-2571321(+)	5.64	70174.95	chlo
JX1.Chr10g00487	sdCRK	Chr10:6490193-6494854(+)	7.42	40054.75	plas
JX1.Chr10g00658	ddCRK	Chr10:9958170-9964266(-)	5.91	72531.94	plas
JX1.Chr10g00659	ddCRK	Chr10:9971626-9974831(-)	6.58	73900.45	plas
JX1.Chr10g00672	ddCRK	Chr10:10128925-10136617(-)	6.81	73357.05	plas
JX1.Chr10g01596	ddCRK	Chr10:20663997-20668313(+)	8.3	66318.14	plas
JX1.Chr11g01436	sdCRK	Chr11:17940319-17956428(-)	8.14	64886.1	plas
JX1.Chr11g02138	ddCRK	Chr11:26246115-26249367(+)	7.16	77221.23	plas
JX1.Chr11g02777	ddCRK	Chr11:32795546-32798576(-)	6	75861.23	plas
JX1.Chr12g02003	ddCRK	Chr12:26580785-26591145(+)	7.51	70624.28	plas
JX1.Chr12g02015	ddCRK	Chr12:26646535-26647878(+)	6	45307.69	extr
JX1.Chr12g02022	ddCRK	Chr12:26732119-26734182(+)	5.64	74407.12	plas
JX1.Chr12g02025	PDLD	Chr12:26744303-26746618(-)	6.61	43417.74	cyto
JX1.Chr12g02027	ddCRK	Chr12:26751216-26752887(-)	5.54	51111.67	E.R.
JX1.Chr12g02246	CRRSP	Chr12:28825689-28828289(+)	10.44	20526.36	chlo
JX1.Chr12g02251	ddCRK	Chr12:28856486-28857833(-)	7.04	34574.8	vacu
JX1.Chr12g02252	CRRSP	Chr12:28859067-28859465(-)	9.3	14005.3	nucl

[1] Note: subcellular localization Chlo: chloroplast; Golg_ Plas: endoplasmic reticulum; Extr: extracellular matrix; Plas: plasma membrane; GOLG: Golgi; E. R.: endoplasmic reticulum; Nucl: Nucleus; Vacuole; Cyto: cytoplasm.

Table S2. Orthologous genes containing DUF26 domain in five Gramineous crops.

Oryza rufipogon Griff.	*Sorghum bicolor (L.) Moench*	*Triticum aestivum L.*	*Zea mays Linn.*	*Zizania aquatica L*
JX1.Chr12g02025	*#N/A*	*TraesCS5A02G052300.1*	*#N/A*	*#N/A*
JX1.Chr05g02169	*KXG22251*	*#N/A*	*Zm00001eb291380_T001*	*GWHTBFHI000462*
JX1.Chr07g01850	*#N/A*	*#N/A*	*#N/A*	*GWHTBFHI035965*
JX1.Chr07g01852	*#N/A*	*#N/A*	*#N/A*	*#N/A*
JX1.Chr07g01872	*#N/A*	*#N/A*	*#N/A*	*GWHTBFHI037529*
JX1.Chr02g02781	*OQU82029*	*TraesCS6B02G286000.1*<br>*TraesCS6D02G220800.1*	*Zm00001eb186090_T001*	*GWHTBFHI021698*
JX1.Chr03g01309	*#N/A*	*#N/A*	*#N/A*	*#N/A*
JX1.Chr07g01849	*EER97337*<br>*EER99490*	*TraesCS2A02G216700.1*<br>*TraesCS2B02G241800.1*<br>*TraesCS2D02G222300.1*	*Zm00001eb323300_T001*	*GWHTBFHI037523*
JX1.Chr07g01881	*#N/A*	*#N/A*	*#N/A*	*GWHTBFHI035947*
JX1.Chr07g01883	*#N/A*	*#N/A*	*#N/A*	*#N/A*
JX1.Chr07g01860	*#N/A*	*#N/A*	*#N/A*	*#N/A*
JX1.Chr07g01880	*#N/A*	*#N/A*	*#N/A*	*GWHTBFHI035948*
JX1.Chr10g00202	*#N/A*	*#N/A*	*#N/A*	*#N/A*
JX1.Chr07g01878	*#N/A*	*#N/A*	*Zm00001eb323300_T001*	*#N/A*
JX1.Chr03g01308	*EER92464*	*TraesCS4A02G099000.1*	*Zm00001eb012570_T001*	*GWHTBFHI015437*

		TraesCS4B02G205300.1<br>*TraesCS4D02G206200.1*		*GWHTBFHI024268*
JX1.Chr07g01876	*EER99493*	*#N/A*	*Zm00001eb323420_T001*	*GWHTBFHI035944*<br>*GWHTBFHI037533*
JX1.Chr04g02176	*OQU82029*	*TraesCS2A02G386500.1*<br>*TraesCS2B02G404100.1*<br>*TraesCS6D02G220800.1*	*#N/A*	*GWHTBFHI017350*<br>*GWHTBFHI018956*
JX1.Chr07g01884	*#N/A*	*#N/A*	*#N/A*	*#N/A*
JX1.Chr01g01821	*#N/A*	*#N/A*	*Zm00001eb131710_T001*	*#N/A*
JX1.Chr07g01853	*EER99485*<br>*KXG36411*	*TraesCS2A02G216400.1*<br>*TraesCS2B02G241500.1*<br>*TraesCS2D02G222000.1*	*Zm00001eb106380_T001*	*GWHTBFHI035963*
JX1.Chr07g01882	*EER97337*	*TraesCS2A02G216700.1*<br>*TraesCS2A02G216900.1*<br>*TraesCS2B02G240900.1*<br>*TraesCS2B02G241800.1*<br>*TraesCS2D02G221400.1*<br>*TraesCS2D02G221500.1*	*#N/A*	*#N/A*
JX1.Chr10g00672	*#N/A*	*#N/A*	*#N/A*	*#N/A*
JX1.Chr07g01886	*#N/A*	*#N/A*	*#N/A*	*#N/A*
JX1.Chr07g01887	*#N/A*	*#N/A*	*#N/A*	*#N/A*
JX1.Chr03g02438	*EER91340*	*#N/A*	*Zm00001eb043080_T001*	*#N/A*
JX1.Chr07g01858	*#N/A*	*#N/A*	*#N/A*	*#N/A*
JX1.Chr07g01877	*#N/A*	*TraesCS2A02G215800.1*<br>*TraesCS2B02G240800.1*	*#N/A*	*#N/A*
JX1.Chr03g01564	*#N/A*	*#N/A*	*#N/A*	*GWHTBFHI015230*
JX1.Chr07g01845	*OQU90047*	*TraesCS2A02G217200.1*<br>*TraesCS2B02G242500.1*<br>*TraesCS2D02G223000.1*	*Zm00001eb323230_T001*	*GWHTBFHI035968*
JX1.Chr04g00767	*#N/A*	*#N/A*	*#N/A*	*#N/A*
JX1.Chr07g01855	*#N/A*	*#N/A*	*#N/A*	*#N/A*
JX1.Chr07g01854	*KXG36419*	*TraesCS2B02G241600.1*<br>*TraesCS2D02G222100.1*	*#N/A*	*GWHTBFHI035962*
JX1.Chr05g00107	*EES18898*	*TraesCS1A02G035600.1*	*Zm00001eb266690_T001*	*GWHTBFHI001334*
JX1.Chr04g00378	*#N/A*	*#N/A*	*#N/A*	*#N/A*
JX1.Chr12g02015	*#N/A*	*#N/A*	*#N/A*	*#N/A*
JX1.Chr12g02022	*OQU79555*	*TraesCS5A02G051200.1*<br>*TraesCS5A02G052400.1*<br>*TraesCS5B02G057205.1*<br>*TraesCS5B02G064400.1*<br>*TraesCS5D02G062100.1*<br>*TraesCS5D02G062800.1*	*Zm00001eb031390_T001*	*GWHTBFHI028861*

JX1.Chr01g01637	*#N/A*	*#N/A*	*#N/A*	*GWHTBFHI004479*
JX1.Chr10g00659	*#N/A*	*#N/A*	*#N/A*	*#N/A*
JX1.Chr07g01885	*#N/A*	*#N/A*	*#N/A*	*#N/A*
JX1.Chr07g01857	*#N/A*	*#N/A*	*#N/A*	*#N/A*
JX1.Chr07g01842	*KXG36392*	*TraesCS2A02G217300.1*<br>*TraesCS2B02G242600.1*<br>*TraesCS2D02G223100.1*	*Zm00001eb323220_T001*	*GWHTBFHI035969*
JX1.Chr07g01869	*KXG36409*	*TraesCS2A02G216300.1*<br>*TraesCS2B02G241400.1*<br>*TraesCS2D02G221900.1*	*Zm00001eb323380_T001*	*GWHTBFHI035959*<br>*GWHTBFHI037525*
JX1.Chr10g00658	*#N/A*	*#N/A*	*#N/A*	*#N/A*
JX1.Chr08g00264	*#N/A*	*#N/A*	*#N/A*	*#N/A*
JX1.Chr11g02138	*#N/A*	*#N/A*	*#N/A*	*#N/A*
JX1.Chr07g01859	*EER99486*	*#N/A*	*#N/A*	*GWHTBFHI035960*
JX1.Chr08g00267	*#N/A*	*#N/A*	*#N/A*	*#N/A*
JX1.Chr11g02777	*#N/A*	*#N/A*	*#N/A*	*#N/A*
JX1.Chr01g02212	*EES00909*	*#N/A*	*Zm00001eb162360_T001*	*GWHTBFHI004789*
JX1.Chr08g00268	*#N/A*	*#N/A*	*#N/A*	*#N/A*
JX1.Chr08g00266	*#N/A*	*#N/A*	*#N/A*	*#N/A*
JX1.Chr07g02769	*#N/A*	*#N/A*	*#N/A*	*GWHTBFHI035473*
JX1.Chr07g01839	*EER99466*	*TraesCS2A02G217600.1*<br>*TraesCS2B02G243100.1*	*Zm00001eb106320_T001*	*GWHTBFHI037520*
JX1.Chr01g02186	*#N/A*	*#N/A*	*#N/A*	*#N/A*
JX1.Chr04g03076	*#N/A*	*#N/A*	*#N/A*	*#N/A*
JX1.Chr12g02027	*#N/A*	*#N/A*	*#N/A*	*GWHTBFHI009052*
JX1.Chr03g00322	*OQU93264*	*TraesCS5A02G511600.1*<br>*TraesCS4B02G342500.1*<br>*TraesCS4D02G338100.1*	*#N/A*	*GWHTBFHI016127*<br>*GWHTBFHI024706*
JX1.Chr10g00487	*#N/A*	*#N/A*	*#N/A*	*GWHTBFHI007906*
JX1.Chr07g02496	*#N/A*	*#N/A*	*#N/A*	*#N/A*
JX1.Chr05g00227	*EES17654*	*TraesCS1A02G073300.1*<br>*TraesCS1B02G091900.1*<br>*TraesCS1D02G076200.1*	*Zm00001eb354860_T001*	*GWHTBFHI001412*<br>*GWHTBFHI032277*
JX1.Chr01g02325	*OQU87032*	*#N/A*	*#N/A*	*#N/A*
JX1.Chr03g01379	*#N/A*	*#N/A*	*#N/A*	*#N/A*
JX1.Chr04g00298	*#N/A*	*#N/A*	*#N/A*	*GWHTBFHI019945*
JX1.Chr07g01840	*#N/A*	*TraesCS2A02G217500.1*<br>*TraesCS2D02G223300.1*	*#N/A*	*GWHTBFHI037521*
JX1.Chr10g00203	*#N/A*	*#N/A*	*#N/A*	*#N/A*
JX1.Chr02g03361	*OQU85453*	*TraesCS6A02G297000.1*<br>*TraesCS6B02G326800.1*<br>*TraesCS6D02G277200.1*	*Zm00001eb253780_T001*	*GWHTBFHI026222*

JX1.Chr01g03700	*#N/A*	*#N/A*	*#N/A*	*#N/A*
JX1.Chr12g02003	*#N/A*	*TraesCS5B02G057500.1*	*#N/A*	*#N/A*
JX1.Chr06g01056	*EER89540*	*TraesCS7A02G202700.1*<br>*TraesCS7B02G109700.1*<br>*TraesCS7D02G206000.1*<br>*TraesCS6D02G277200.1*	*Zm00001eb280720_T001*<br>*Zm00001eb371540_T001*	*GWHTBFHI007139*<br>*GWHTBFHI034762*
JX1.Chr04g01126	*KXG26129*	*#N/A*	*Zm00001eb421270_T001*	*#N/A*
JX1.Chr07g00831	*#N/A*	*#N/A*	*#N/A*	*#N/A*
JX1.Chr11g01436	*#N/A*	*#N/A*	*#N/A*	*#N/A*
JX1.Chr07g01841	*KXG36391*	*TraesCS2B02G243000.1*	*Zm00001eb323200_T001*	*GWHTBFHI037522*
JX1.Chr01g02331	*#N/A*	*#N/A*	*#N/A*	*#N/A*
JX1.Chr10g01596	*OQU91617*	*TraesCS1A02G159500.2*<br>*TraesCS1B02G175900.3*<br>*TraesCS1D02G156900.1*	*Zm00001eb221490_T001*	*GWHTBFHI008457*
JX1.Chr07g01889	*#N/A*	*#N/A*	*#N/A*	*#N/A*
JX1.Chr01g03703	*#N/A*	*TraesCS3A02G320500.1*	*#N/A*	*#N/A*
JX1.Chr12g02246	*#N/A*	*#N/A*	*#N/A*	*#N/A*
JX1.Chr12g02251	*#N/A*	*#N/A*	*#N/A*	*#N/A*
JX1.Chr07g01847	*KXG36398*	*#N/A*	*Zm00001eb106370_T001*	*GWHTBFHI035967*
JX1.Chr07g01846	*#N/A*	*#N/A*	*Zm00001eb106370_T001*	*#N/A*
JX1.Chr07g01848	*#N/A*	*#N/A*	*#N/A*	*#N/A*
JX1.Chr01g03688	*#N/A*	*#N/A*	*#N/A*	*#N/A*
JX1.Chr12g02252	*#N/A*	*#N/A*	*#N/A*	*#N/A*
JX1.Chr01g02330	*#N/A*	*#N/A*	*#N/A*	*#N/A*
JX1.Chr01g03687	*#N/A*	*#N/A*	*#N/A*	*#N/A*

Table S3. Expression of DUF26 domain-containing genes and submergence tolerance genes with known function in DXWR under submergence stress.

ID	CK-2d_fpkm	CK-4d_fpkm	AG-2d_fpkm	AG-4d_fpkm	SS-0d_fpkm	SS-3d_fpkm	SS-5d_fpkm
JX1.Chr03g01308	24.445	41.375	809.08	50.88	8.01	196.035	193.655
JX1.Chr03g01309	2.72	1.86	497.975	10.44	0.975	13.125	4.7
JX1.Chr03g01564	0.345	4.9	222.045	10.665	0	0	0.035
JX1.Chr04g00767	41.255	48.42	1390.72	109.85	10.29	32.635	20.52
JX1.Chr04g03076	40.34	370.225	3564.35	71.605	11.25	144.85	204.37
JX1.Chr06g01056	14.275	12.945	5.185	5.36	4.88	4.67	4.535
JX1.Chr07g01880	6.215	4.52	0.68	0.385	2.33	0.175	0.2
JX1.Chr07g02769	0.805	0.13	12.41	2.13	0	0	0
JX1.Chr08g00264	47.17	41.61	11114.74	855.145	0.33	0.475	0.6
JX1.Chr08g00266	2.93	1.94	616.455	22.86	0.03	0.13	0.185
JX1.Chr08g00267	38.825	68.135	7659.885	195.29	0.06	0	0
JX1.Chr08g00268	42.8	57.69	12631.365	246.025	0.455	0.335	0.29

JX1.Chr07g01842	4.55	1.645	20.395	5.985	1.245	1.055	1.745
JX1.Chr07g01853	19.395	28.255	62.72	47.79	7.425	29.38	29.45
JX1.Chr07g01854	7.88	12.03	33.76	30.79	3.225	12.9	11.08
JX1.Chr07g01855	8.855	6.42	43.365	24.445	0.79	2.435	3.835
JX1.Chr07g01857	25.73	27.285	54.78	51.96	2.02	43.66	45.09
JX1.Chr07g01858	5.24	5.445	20.19	8.695	0.23	1.11	1.805
JX1.Chr07g01859	23.265	30.7	36.04	53.865	5.7	41.56	43.725
JX1.Chr07g01860	9.205	9.87	22.665	33.19	1.72	13.34	14.44
JX1.Chr07g01869	3.845	8.96	9.155	2.895	5.7	13.665	12.965
JX1.Chr07g01881	6.745	4.87	1.065	1.655	2.14	1.2	1.69
JX1.Chr07g01882	0	0.295	0	0	3.045	1.555	1.83
JX1.Chr07g01884	0.25	0.53	23.045	13.09	1.58	15.03	15.435
JX1.Chr07g01885	9.97	8.115	32.035	22.45	9.11	17.205	18.335
JX1.Chr07g01887	15.675	31.94	10.06	14.005	21.905	97.77	80.255
JX1.Chr07g01889	11.915	6.475	16.28	21.455	1.595	17.065	19.74
JX1.Chr07g02496	0.205	2.865	0.015	0.055	25.02	5.445	5.93
JX1.Chr10g00202	0	0.03	0.01	0	1.035	0.265	0.36
JX1.Chr10g00659	0.185	0.145	4.255	1.15	0.37	1.69	1.685
JX1.Chr11g02777	0	0.08	0	0.165	0.195	3.615	3.06
JX1.Chr01g01821	1.9	0.825	9.475	4.055	0.18	0.21	0.3
JX1.Chr05g00227	5.335	6.355	1.785	3.315	1.69	9.05	10.915
CIPK14	16.95	22.67	202.685	129.045	31.39	75.58	101.085
CIPK15	24.62	32.41	180.855	174.59	39.775	94.915	125.715
SnRK1A	43.515	42.9	41.38	80.72	25.115	31.2	33.485
MPK3	46.185	59.495	54.755	31.665	10.1	31.03	27.95
OsERF67	103	88.295	118.87	139.75	68.385	135.43	163.22
OsERF66	2.405	1.13	12.68	3.23	0.335	3.745	3.045
SLR1	167.425	224.615	97.805	37.705	128.59	136.695	115.84
SLRL1	10.48	9.735	16.955	17.165	5.475	7.865	8.675
RAmy3D	0.215	4.175	178.115	1.095	1.305	1.645	1.67
G3PDH	556.905	348.465	4794.855	1953.99	55.8	124.78	98.04
PPDK	8.1	2.36	788.045	628.65	1.755	37.65	38.63
